# Refugee and migrants' involvement in participatory spaces in a US practice‐based research network study: Responding to unanticipated priorities

**DOI:** 10.1111/hex.13764

**Published:** 2023-04-20

**Authors:** Joseph W. LeMaster, Cory B. Lutgen, Jagtaj Matharoo, Anne E. MacFarlane

**Affiliations:** ^1^ University of Kansas School of Medicine Kansas City Kansas USA; ^2^ American Academy of Family Physicians National Research Network Leawood Kansas USA; ^3^ School of Medicine, Faculty of Education and Health Services University of Limerick Limerick Ireland; ^4^ School of Medicine, Health Research Institute, Faculty of Education and Health Services University of Limerick Limerick Ireland

**Keywords:** communication barriers, community‐based participatory research, culturally competent care, health services accessibility, refugees and migrants

## Abstract

**Background:**

Refugees and migrants face suboptimal involvement in spaces for primary healthcare decision‐making. Given the rising numbers of resettled refugees and migrants in primary care settings in the United States, there is an urgent need for patient‐centred outcome research in practice‐based research networks (PBRNs) with diverse ethnolinguistic communities. This study explored whether researchers, clinicians and patients would achieve consensus on (1) a common set of clinical problems that were applicable across a PBRN and (2) potential clinical interventions to address those problems to inform a patient‐centred outcomes research (PCOR) study in a similar research network.

**Methods:**

In this qualitative participatory health research study, patients from diverse ethnolinguistic communities and clinicians from seven practices in a US PBRN discussed preferences for PCOR responsive to patients and the clinicians who serve them in language‐discordant settings. Researchers and an advisory panel that included patients and clinicians from each participating practice held regular advisory meetings to monitor progress on project milestones and solve emerging problems. Participants took part in 10 sessions using Participatory Learning in Action and the World Café methods to identify and prioritise their ideas, using questions set for them by the advisory panel. Data were analysed based on principles of qualitative thematic content analysis.

**Results:**

Participants identified common barriers in language‐discordant healthcare settings, principally patient‐clinician communication barriers and suggestions to overcome these barriers. A key finding was an unanticipated consensus about the need for attention to healthcare processes rather than a clinical research priority. Negotiation with research funders enabled further analysis of potential interventions for care processes to improve communication and shared decision‐making in consultations and the practice as a whole.

**Conclusion:**

PCOR studies should examine interventions for improving communication between patients from diverse ethnolinguistic communities and primary care staff if the sorts of harms experienced by patients experiencing language‐discordant healthcare are to be reduced or prevented. Flexibility and responsiveness from funders to unanticipated findings are key structural supports for participatory health research in primary care clinical settings with this population and others who experience marginalisation and exclusion.

**Patient or Public Contribution:**

Patients and clinicians participated in the study both in the formulation of the study question, data collection, analysis and dissemination of these results; consented to their individual participation; and reviewed early drafts of the manuscript.

## 1 INTRODUCTION

The World Health Organisation has developed primary care policies that promote the involvement of patients and communities in health decision‐making. These policies emphasise the need for people's involvement as individuals (e.g., in their consultations) and as communities (e.g., in service development, research and policymaking).^1^, ^2^ The rationale underpinning these policies is twofold: people have a right and responsibility to be involved *and* their involvement can lead to a more comprehensive knowledge base to guide the delivery of responsive, person‐centred healthcare. Taken together, people's involvement in health decision‐making can promote health equity.^3^, ^4^

Shared decision‐making in consultations, community advisory boards for research, and involvement in mechanisms for policymaking may all be conceptualised as participatory spaces in contemporary primary healthcare.^5^ By definition, participatory spaces are shaped by physical, temporal and social dimensions that influence the opportunity for, and enactment of health decision‐making.^6^

For example, whether brief interactions occur in primary care practices focused on clinical goals; or in community settings with lengthy meetings focused on research or policy goals shapes the scope for involvement in health decision‐making. It can, however, be challenging for service providers to adjust to new social identities beyond their caregiving role as they move from the consultation space to those set up for research or service development projects^7^; and to accept the time required for work in community settings.^8^ Thus, participatory spaces in primary healthcare share an *implementation problem*—typically they are not embedded as routine and normalised practice in primary healthcare.^9^ Further, a *pattern of exclusion* whereby patients and communities who experience marginalisation experience suboptimal involvement may be revealed in each of these participatory spaces.^10^, ^11^

Given contemporary global migration patterns, refugees and other migrants in the United States and other resettlement countries bring increasing cultural and linguistic diversity to primary care settings, such that cross‐cultural consultations are no longer confined to specialist refugee clinics but are increasingly a feature of mainstream clinical practice even in rural and remote areas.^12^, ^13^ There is thus an urgent need to attend to the suboptimal involvement of refugees and migrants in primary healthcare decision‐making; to understand their health needs and to take action for equitable health outcomes. Yet, despite the policy and legal imperative, variability in the quality of care for migrants persists. The unavailability in the use of trained interpreters in consultations (in‐person or by telephone) leads to inappropriate diagnoses and treatments, medication errors, higher emergency department use, and longer hospitalisations.^14^ Despite the mandatory provision of language interpretive services in US primary care settings that care for patients with publicly provided health insurance (Medicare, Medicaid),^15^ and further clear evidence for inferior outcomes when providers are linguistically discordant from patients,^16^, ^17^, ^18^, ^19^ bilingual clinicians and clinical staff are still the exception rather than the rule, so that US patients with limited English proficiency experience limited access and care quality.^20^ Further, in the US, patient‐centred outcomes research (PCOR) with refugees and migrants in practice‐based networks has been challenging. PCOR focused on various medical conditions including diabetes, asthma and gout found unique relational and communication issues relevant to different individual cultural groups at individual sites—which make involvement with these populations daunting logistically.^21^, ^22^, ^23^, ^24^, ^25^, ^26^, ^27^, ^28^, ^29^, ^30^, ^31^, ^32^ This has led to culture‐specific approaches to PCOR with such patients, each individualised to the culture in a particular project. This is valuable for the local setting but means that data and interventions are not easily comparable or generalisable across healthcare systems, thus limiting the scope for systemic change more broadly in primary care services.^21^, ^22^, ^23^, ^24^, ^25^, ^29^, ^30^, ^31^, ^33^, ^34^

In the United States, practice‐based research networks (PBRNs) are an essential mechanism to deal with these issues because they are designed to involve patients and community members from multiple practices in PCOR. This paper explores the involvement of refugees and migrants from diverse ethnolinguistic populations who do not have a shared language and cultural background with their primary care providers, whose practices participate in a nationwide US PBRN. In line with international evidence,^35^ these populations have not been well represented in PBRN activities before this project. The current study was designed to engage multiple refugees and migrant patient groups in these practices to identify clinical problems that need attention across groups; and to develop a broad‐based, well‐interfaced network of patient‐engaged primary care clinics ready to move forward together to conduct comparative effectiveness studies to address the clinical problem they identified as most important. Although the primary goal of the funded project was to generate an operational guide for future PCOR in PBRNs with diverse ethnolinguistic groups,^36^ a secondary goal was to seek consensus across a PBRN on a clinical research project, since consensus is an important indicator of *shared decision making* given the power asymmetries between patients and practice staff.^37^

The overall aim of this paper is to explore patient and clinician perspectives during that process. Specific objectives are to examine whether researchers, clinicians and patients across the emerging network would achieve consensus on (1) a common set of clinical, that is, health problems that were applicable across participating primary care practices, and (2) potential clinical interventions to address those problems to inform a PCOR study in a similar context. We employ the concept of participatory space as a heuristic device to describe methods, interpret findings and make recommendations.

## 2 METHODS

### 2.1 Study context

This research is based on a 24‐month project funded by the US funder Patient‐centered Outcomes Research Institute that focused on refugees, other migrants and primary care providers who did not have a shared language or cultural background. The project was designed as a participatory research project and had ethical approval from the American Academy of Family Physicians Institutional Review Board. The project involved primary care practice staff, refugees and migrants from multiple primary care clinics who had experience in cross‐cultural communication and a shared interest in optimising health outcomes for refugee and migrant health.

The project had a central organising team of two network leaders (J. W. L., C. L.), whose role was to monitor project milestones and progress; and to support methodology and logistics at all project sites.

This team worked with a project advisory board composed of practice clinicians and patient representatives and an international advisor with a social science background (A. E. M.). A medical student (J. M.) joined the team in 2020 to gain research experience and support qualitative data analysis.

### 2.2 Sampling and recruitment/retention of participants

The clinicians and patients in the project are the samples for the analysis on which this paper is based. Based on principles of purposeful sampling for information‐rich participants,^38^ all network primary care practices that serve linguistically and culturally diverse patient populations were invited to participate in the project. Those who opted in took part from the inception of the project, including its initial design. In total, seven primary care practices participated from six US states. Practices varied in size (solo to many providers), language preference of patients served and approaches to supporting patients' language needs (in‐person vs. phone interpreters vs. bilingual staff). In participating practices, at least 25% of patients were from diverse ethnolinguistic groups.

There were no exclusion criteria for healthcare providers at the participating clinics. Twenty‐three clinicians at the seven practices agreed to take part.

Each practice nominated a Site Coordinator and a Lead Clinician (physician) for the project. The Site Coordinator was a practice manager or lead nurse who served as the point of contact between the clinic and the project organising team, as well as with the patients, caregivers or practice staff who took part.

In the first month of the project, to facilitate communication with clinicians and participating patients from different ethnolinguistic groups, Site Coordinators invited at least five patients from each practice who were refugees or migrants and who were bilingual in English to participate in group discussions. Patients were informed that participation was voluntary and that the provision of their medical care would be unaffected by their decision to participate (or not). We did not collect personal health information or ethnolinguistic data from individual participants because the emphasis was on collective experiences and collective knowledge generation at each practice site.

We excluded patients who could not participate for cognitive reasons. Participating patients received usual care from any primary care physician or other clinicians in the practice, including nurse practitioners or physician assistants.

To maximise practice and patient retention, we used published recommendations for minimising losses to follow‐up that we have used successfully in prior studies.^39^, ^40^, ^41^, ^42^ For practices, we used twice monthly email interaction and/or online meetings. One site withdrew from further participation after session 4 citing their own lack of staff capacity as the reason for doing so. Data provided by that practice before session 5 were included for analysis.

For patients, Site Coordinators collected multiple types of contact information from participating patients/caregivers—telephone (including cellular), mailing address and email addresses for the patients and alternate contacts. We incentivized patient engagement and retention with $30 USD payments for participation in each site‐based discussion. Overall, while not every clinician and patient attended every session, the attendance records at each clinical site showed strong retention of most participants across the sessions. Table 1 describes practices, the number of providers and ethnolinguistic communities served by the practice.

**Table 1:** Practice characteristics.

Practice	Participating practice staff (#)^a^	Participating patients (#)^a^	Patient ethnolinguistic groups (practice‐wide)^b^
A	2	7	Latin
B	5	10	Afghan, Arab, DRC, Rwanda, Burundi, Burma, Somali, Bhutanese, and others
C	3	5	Somali, East African, Latin
D	2	7	Burmese, Bangla, Karen, Nepali, Arabic, Swahili, Somali, Karenni, Kinyarwanda, Tigrinya, French, Persian, Vietnamese
E	5	6	Amharic, Arabic, Nepali, Dari, Russian, Somali, Tigrinya, Burmese, others
F	4	5	Latin, Burmese, Nepali
G^c^	2^c^	5^c^	Data Unavailable^c^

^a^ Participating practice staff included physicians, nurses, nurse managers, and other clinic programme personnel. Data for participating practice staff and patients represent the total number of practice staff or patients that participated over the course of the sessions. Not every patient or practice staff participated in every session.

^b^ Ethnicities presented represent ethnicities or the language of patients treated across the entire clinic and are not representative of individual ethnicities of study participants, which was not reported.

^c^ Practice G withdrew from study participation after session 4. Data regarding patient ethnicities is not available.

### 2.3 Data generation and analysis

We used a blended approach for data generation and coanalysis by combining two participatory approaches that have been effective in qualitative primary care research with refugees and migrants.^43^, ^44^, ^45^ We integrated principles from Participatory World Café (PWC) and Participatory Learning in Action (PLA) methods. PWC and PLA are similar group discussion/interaction methods designed to address power asymmetries in participatory spaces between diverse participants.^46^ Both methods are designed to bring diverse collaborators from different types of organisations together to generate qualitative data; emphasise ‘real‐time’ coanalysis of emerging themes^47^; and include prioritisation of the ideas generated in the discussion to guide concrete action/next steps.^44^, ^48^ The US‐based organising team was responsible for logistical and methodological oversight of the process. Site coordinators and leaders initially came to Kansas City for a 1‐day training in which we practised data generation methods to support their consistent use across the seven participating clinical sites. The topic guide for data generation was developed iteratively by the Advisory Board during the project based on initial and emergent areas of investigation (see Supporting Information: Appendix I).

### 2.4 PWC method

Using this method, site leaders, coordinators and bilingual patients at each site discussed a series of broad, interrelated questions in small groups about things they wanted to see changed to improve their health, for example, health problems, communication with the practice, healthcare access, and communication in consultations. Participants brainstormed answers to each posed question and recorded their responses on a poster board. This produced a list of responses to each question, which participants then prioritised by voting for their top three responses to each question. Each participant could allocate their three votes as desired (i.e., all votes for a single response or across separate responses). This process proceeded until all posed questions was discussed.^49^

### 2.5 PLA

During subsequent sessions, PLA methodology was used where participants initially engaged in a Flexible Brainstorm exercise about the issues under investigation.^48^ They individually, nonverbally brainstormed as many responses as they could think of to a single question, writing each response on a separate sticky note. Each participant then, in turn, explained each response to other group members. After sharing their idea, participants placed them on a central poster board, in the centre of which the posed question was written. They placed their idea on a sticky note nearer to or further from the question physically, based on their own judgement of how impactful it was to address the question. Nearer indicated ‘maximally impactful’ and further away indicated less impactful. During this stage, participants could generate additional responses, if any new ideas occurred to them (hence ‘flexible’ ‘brainstorming’). After all participants shared all their ideas, participants collectively engaged in a card sort.^48^ For this, they rearranged their ideas into emerging themes, so that responses that were more closely related were physically grouped together; and so that those themes which participants collectively perceived to be most impactful to the question were closest to the question on the board. After group members agreed on the rearrangement, they then identified a title for each theme they had created together and labelled the theme on the board.

**Figure 1:**
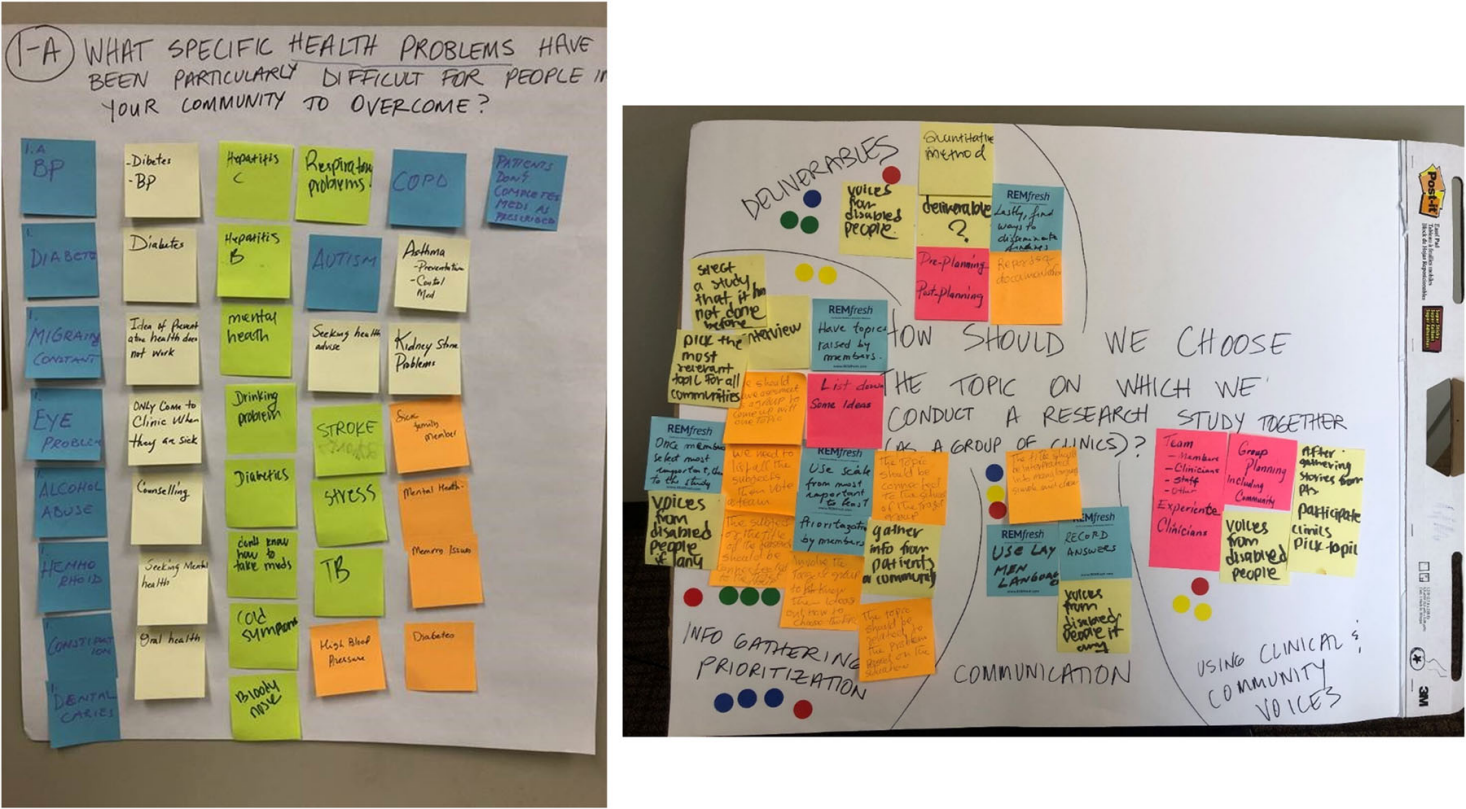
Sample World Café (L) and Participatory Learning in Action (R) discussion board results.

Figure 1 shows representative discussion boards from both types of sessions (PWC and PLA). Photographs of all discussion boards are available upon request.

We collected data during 10 such sessions. To support data trustworthiness, alternating months (when we did not conduct site‐based discussions with patients), the US‐based organising team, two patient representatives and up to two clinical staff representatives from each site (always including either the Lead Physician or Site Coordinator from each site) met virtually to reflect on the incoming data and themes that had been identified; and (based on those) to iteratively decide upon the questions to be asked at the succeeding site‐based discussion.

After all data were collected, the author group examined the data iteratively using a thematic content analysis approach.^47^ Three coders (J. W. L., C. L., T. M.) independently reviewed the data and its responsiveness to study questions, for example, exploring whether/how participants would identify a common set of barriers and facilitators applicable across participating primary care practices to conduct PCOR that is responsive to both clinician *and* patient preferences. The purpose of this analysis was to ensure that the iterative analysis conducted in between data generation sessions was complete and synthesised to understand anticipated and unanticipated emergent findings (described in detail in Section Results, 3). Coders generated memos elucidating these codes during this analysis process, which were reviewed and confirmed by consensus between the three coders. Final memos were reduced and organised into seven overarching cross‐cutting themes, using a similar consensus approach. A fourth researcher who was not involved in the data collection or initial analysis (A. E. M.) reviewed the data, the original list of memos and the final cross‐cutting themes to confirm that the data matched the codes. An early draft of this manuscript was circulated to site leaders to obtain member checks from participants and ensure that the cross‐cutting themes were consistent with their own perceptions about the project (which they indeed were). The final seven cross‐cutting themes are listed in Box 1.

Box 1: **Themes**

1) Seeking consensus on a common set of clinical problems that were applicable across participating primary care practices
2) Language barriers
3) Doctor‐patient cross‐cultural communications
4) Lack of awareness and sensitivity to a patient's culture
5) Lack of access to healthcare appointments
6) Problems with medication adherence
7) Consensus on potential clinical interventions to address these problems

In Section Results, 3, we present key findings in relation to the two objectives of this paper: can researchers, clinicians and patients seek consensus on (1) a common set of clinical problems that were applicable across participating primary care practices and (2) potential clinical interventions to address those problems to inform a research grant application.

## 3 RESULTS

The following themes were identified in our analysis. Please also see Table 1, in which we identify participating practices by letters (A–G). We refer below to the practices from which comments related to each theme originated.

1. *Seeking consensus* on a common set of clinical problems that were applicable across participating primary care practices. In terms of the frequency of health problems cited, in the initial session sites most commonly identified diabetes mellitus followed by chronic stress and obesity as the health problems most difficult for their communities to overcome. It became very clear from as early as session 2, however, that the majority of problems identified by patients and clinicians related to care processes such as barriers to accessing care and barriers to high‐quality care rather than to those health problems. These are presented below by theme based on combined data from patients and clinicians at each site, according to priority. Citations note the site that contributed to the reported perspective (sites A–G).
2. *Language barriers* were the most highly prioritised themes by participants. Barriers included having to wait extensive time for an interpreter during an appointment, incorrect interpretation of information to patients, and the unavailability of patient resources, for example, written materials in patients' preferred languages [C,D]. Participants also noted that lack of skill and/or knowledge of correct interpretation technique by interpreters caused miscommunication, for example, messages left via voicemail, not in the patient's preferred language [B], messages given to children answering phones, and calls made by health‐system staff at inconvenient times [D]. Other sites mentioned the importance of clinicians not knowing how to use an interpreter correctly [D,E,F].
3. The next prioritised theme referred to *doctor–patient cross‐cultural interactions*. During appointments, clinicians often lacked an understanding of the patient's culture (and vice versa) [C,E]. This was problematic when bilingual clinicians overestimated their fluency in a language, causing a patient to misinterpret what was going on in the appointment [F]. This contributed to problematic power dynamics as patients didn't want to appear ignorant or offend the provider, so they would not speak up, even if they didn't understand [F]. In addition, patients misunderstood diagnoses made and medications prescribed and felt dehumanised by lack of education provided [D,E], or else by information overload [D]. They reported frustration with clinicians who asked them to make healthcare decisions immediately during the appointment without giving them time to discuss the decision with their families [F]; and wanted more time in appointments [C]. Per the participants, clinicians must demonstrate empathy and respect, patiently let the patient explain fully their reasons for being there, make eye contact (though this differed by patient ethnic group, for some groups direct eye contact implied disrespect), speak using simple words, notice when patients have not ‘bought into’ the discussion [A], use clarifying explanations [G] and focus on interacting with patients rather than the computer [E].
4. A related theme was *awareness and sensitivity to a patient's culture* and gestures, literacy levels in a patient's own language, body language and beliefs around ‘what is appropriate medical care’ [A], as well as trauma and stigma surrounding mental health problems [F,G]. People from different cultures need different responses [D,E,F], including the offer to include traditional healers [D,E]. Clinicians should also pay attention to their own implicit biases and body language; avoid being judgmental and acknowledge that their first encounter with the patient may be the first time a patient has ever seen a doctor [F]. There should be training on providing trauma‐sensitive care [F], meetings about mental health led by language concordant counsellors, translation of medical documents into patients' languages using ‘plain language’ explanations, more education to patients about voicing their concerns as well as on how to deal with stigma for diagnoses such as substance abuse [A,E] and follow‐up on patient connections to community resources [F].
5. Next, participants prioritised the *lack of access to healthcare appointments*. Topics prioritised highly included: unavailability of walk‐in appointments and difficulty with communication about health insurance and coverage limitations [B,D], long wait times at appointments with clinicians [G], difficulties using public transportation to get to appointments [E], unwelcoming responses from the reception staff, as well as frustration when patients must see another clinician whenever their usual primary care provider is unavailable [D]. Patients want the appointment to discuss affordable housing and community support groups, how insurance works and which insurance providers are available [A] and especially how to get help with mental health problems [G]. Safety concerns were also mentioned if nursing staff need to triage patients who lost their insurance and thus could not obtain care [F].
6. Participants, in addition, prioritised a theme *concerning problems with medication adherence*. One site indicated that inadequate information is provided about how to obtain over‐the‐counter medications, how to take a prescribed medication or what the medication is being prescribed for, resulting in incorrect administration or not taking the medication at all [B,D]. With that comes a misunderstanding of one's illness and how the medications work to help [A,C]. Another mentioned that pharmacists should be educated to slow down and use interpreters to explain the medications to the patients, as well as what is covered by insurance [G]. Another site added that discussion was needed as well about how refills work and why there are delays during the restocking of medicines—this will eliminate the fear of taking medications [F].
7. Seeking consensus on potential clinical interventions to address those problems to inform a research grant application.

In several sessions, sites were presented with different examples of clinically oriented interventions such as ones to improve patient perception of chronic pain, and another to promote uptake of colon cancer screening. As early as the second session, however, participants insisted *on revisiting their concerns about care processes*, rather than identifying and developing consensus around PCOR interventions focused on a disease/condition‐related or screening outcome. This caused some discomfort in the project organising team who had a role to monitor project milestones and progress as per the original goals. Nevertheless, in keeping with the participatory ethos of the project and the central tenant underlying PWC and PLA, the organising team understood the importance of hearing the message from clinicians and patients and the need to follow the unanticipated line of inquiry in depth. They liaised with the funders to update them on developments and proceeded to explore care processes rather than clinically oriented research. Therefore, in session 5, participants were presented with an alternative intervention that focused on improving communication between migrants and primary care providers. This was the European RESTORE project, which took place in primary care clinics in 5 European countries.^50^ RESTORE engaged clinicians, migrant patients, and language interpreters in participating clinics during multiple sessions over approximately 12–14 months. It used PLA methods, similar to those used in this study, to support stakeholder collaboration to select a communication guideline or training initiative to implement in their shared clinical setting. A RESTORE‐like project made sense to our participants, so long as clinicians, practice staff and patients were all oriented, trained and supported, everyone's primary skill sets were used, and time and clinic workflow constraints were honoured [A,C,F]. One site perceived that a RESTORE‐style project would require more clinician time than they could afford to give, given the multiplicity of studies already taking place at their clinic[E]. Another site reported ‘The community is anxious for change and would buy in and bring others to the table. Our providers are also eager to make provider‐facing changes to improve communication between providers and patients' [A]. In the end, researchers and sites agreed that interventions implemented in future PCOR studies in this context, for example, primary care clinics with language discordance should focus on patient‐clinician communication rather than clinical outcomes. Thus, at the end of the project, there was consensus on a research focus but one that was a marked departure from the originally anticipated aim of the project. Participants almost unanimously preferred intervention in care processes rather than clinical or health outcomes.

## 4 DISCUSSION

### 4.1 Summary of key findings

This participatory approach to developing a PBRN to progress clinical PCOR in the United States did not reach the desired consensus about a clinical research focus. Instead, it reached an unanticipated consensus about the need for interventions to better care processes. Persisting problems with cross‐cultural communication in primary care consultations and in the wider practice setting were deemed more important by refugees and migrants as well as practice staff. They arrived at an agreement with the central organising team and funders that these problems with care processes warranted further investigation in a PBRN via the implementation of interventions that focused on improving communication between migrants and primary care providers.

### 4.2 Connections with the literature

Findings about persistent problems with cross‐cultural consultations in primary care are consistent with previous studies in the United States and international settings.^16^, ^17^, ^19^, ^51^, ^52^, ^53^ This means that goals for shared decision‐making and person‐centred care for this patient group in the consultation space remain elusive.^9^ Similarly, findings about challenging relational dynamics in the broader practice setting compromise what Kovandzic et al. refer to as the space of access.^51^ The recursive relationship between spaces means that it is valuable to empirically examine them vis‐a‐vis each other to understand how they may mutually constrain or enable access to care.^54^

In this study, there was a shared understanding between patients and the other decision‐makers (service providers, the research team and funders) in these participatory spaces about problems in primary care and the need for interventions in care processes to address them, which is not always the case.^55^, ^56^ Overall, a key message from this participatory primary care research is that those communities who have experienced such marginalisation and exclusion in the clinical setting *really need to be listened to* and *heard*. From an equity perspective, and in line with the participatory health research paradigm in which participation is the central premise, the process of listening and hearing is more important than reaching a priori project objectives, for example, the development of a clinical research study question.^57^ Significantly, in this project, when problems with communication that undermine culturally sensitive, person‐centred care research came to the fore, funders supported the research team to move from the original focus on clinical research interventions. This provided important structural support for the participatory process. The funders' flexible and responsive approach to findings from PWC and PLA can be regarded as respect for decolonising methodologies and new ways of knowing.^58^ A more rigid response would have constrained knowledge production and co‐learning.

There was considerable investment in designing an inclusive, participatory research space. The decision to employ PWC and PLA as innovative and interactive material practices to manage and support power asymmetries in these spaces is still relatively rare.^46^ More standard methods (interviews, focus groups) dominate the field.^59^ The use of PWC and PLA was strongly endorsed by the project participants,^36^ which is in line with findings from previous primary care participatory studies with refugees and migrants in the United States and European contexts.^44^, ^60^, ^61^ It is important to learn how and whether these methods can be scaled up or normalised in primary care research, and whether their use improves measurable health and healthcare outcomes important to patients and clinicians. Other innovative methods, such as arts‐based, culturally attuned, whole‐person methodologies should also be explored to expand the suite of material practices to manage and support power asymmetries in intercultural research spaces.^62^

### 4.3 Methodological critique

The participatory approach in this PBRN‐based project was strong and in line with the hallmarks of quality academic‐community partnerships with community involvement in developing a research question, governance, data interpretation and dissemination.^63^

All participants opted into the project and, thus, we cannot claim representativeness for wider practice or patient populations. Five out of six practices sustained engagement in project activities and dissemination over a duration of 24 months.

The cogeneration and analysis of PWC and PLA data was a strength of the study based on their inherent visual and analytic characteristics that gave voice to patients from diverse ethnolinguistic communities.^61^ We believe that the richness of dialogues within and across sites over time deepened our understanding of the issues raised and provided a unique perspective on the field of migrant healthcare. A limitation is that data generated and analysed collectively are not attributable to individual participants and do not generate extensive quotes in the way that other methods such as interviews or focus groups do. The qualitative analysis during, and after, the project ended adhered, however, to a recognised analytic approach. While the former benefited from participants' insights for data interpretation, site participants were not involved in the final retrospective analysis. Finally, patient participants were bilingual in English and their mother tongue, so were not currently experiencing language discordance with their healthcare clinicians; however, all patient participants had at some time in the past experienced language discordance. Data reported herein represent reports from community representatives about extant language discordance experiences in primary care contexts, rather than participants' current personal health care experiences.

## 5 CONCLUSION

Refugee and migrants' meaningful involvement in a participatory research space using innovative and interactive methods was essential to understand their unanticipated priority: to implement interventions to improve their meaningful involvement in other participatory spaces; that is, the clinical consultation space and the space of access. This is exactly the sort of intervention that PCOR studies should undertake if the sorts of harms experienced by patients experiencing language‐discordant healthcare are to be reduced or prevented. Flexibility and responsiveness from funders to unanticipated findings are key structural supports for participatory health research in primary care clinical settings with this population and others who experience marginalisation and exclusion.

## AUTHOR CONTRIBUTIONS

Joseph W. LeMaster acquired funding for the project leading to the manuscript. Both Joseph W. LeMaster and Cory B. Lutgen conceptualised the project, devised the project methodology and undertook the project administration and data collection and curation. They were joined by Anne E. MacFarlane and Jagtaj Matharoo in the conceptualisation of data analysis, in undertaking that analysis and in writing the original draft and all subsequent edits.

## CONFLICT OF INTEREST STATEMENT

The authors declare no conflict of interest.

## ETHICS STATEMENT

The study was approved by the American Academy of Family Medicine Institutional Review Board.

## Supporting information

Supporting information.

## Data Availability

Data from the study are summarised in the appendices and available in full upon request from the authors. No material (copyrighted or otherwise) was reproduced from other sources. This is not a clinical trial.
