# Supplementary material for: Refugee and migrants' involvement in participatory spaces in a US practice‐based research network study: Responding to unanticipated priorities
Source: Health Expect. 2023 Apr 20;26(4):1596–605. doi: 10.1111/hex.13764 (PMC10349241; doi:10.1111/hex.13764)
Supplement: Supplementary file 1 — Supporting information. [file HEX-26--s001.docx]

Appendix 1. Topical Focus of Discussion Sessions

| **Session #** | **Topic(s)** |
| --- | --- |
| **1** | Community health and healthcare problems & challenges |
| **2** | Patient-clinician communication; Cultural competence; Patient-involved and -centered research; |
| **3** | How to choose a research focus |
| **4** | Community healthcare problems & challenges (revisited) |
| **5** | Implementation of a research intervention (RESTORE example) |
| **6** | Discussing potential research foci |
| **7** | Research outcome measures |
| **8**  **(Pre-Covid)** | Revisiting shared decision-making about a specific research focus |
| **8**  **(Post-Covid)** | Community health problems & challenges revisited- changes during COVID? |
| **9** | Writing a research proposal: role, collaboration, and limitations of patients and clinicians |
| **10** | Discussing possible ‘next step’ interventions |
|  |  |
